# Supplementary material for: High performance data integration for large-scale analyses of incomplete Omic profiles using Batch-Effect Reduction Trees (BERT)
Source: Nat Commun. 2025 Aug 2;16:7104. doi: 10.1038/s41467-025-62237-4 (PMC12318123; doi:10.1038/s41467-025-62237-4)
Supplement: Supplementary file 1 — Supplementary Information [file 41467_2025_62237_MOESM1_ESM.pdf]

High Performance Data Integration for Large-Scale Analyses of Incomplete *Omic* Profiles Using Batch-Effect Reduction Trees (BERT)

Yannis Schumann[1, a, †], **Simon Schlumbohm**[2, †], **Julia E. Neumann**[3, 4, a, *], **Philipp Neumann**[1, 5, a, *]

[1] *Deutsches Elektronen-Synchrotron DESY, Hamburg, Germany*
[2] *Chair for High Performance Computing, Helmut-Schmidt-University Hamburg, Hamburg, Germany*
[3] *Center for Molecular Neurobiology Hamburg (ZMNH), University Medical Center Hamburg-Eppendorf (UKE), Hamburg, Germany*
[4] *Institute of Neuropathology, University Medical Center Hamburg-Eppendorf (UKE), Hamburg, Germany*
[5] *High Performance Computing & Data Science, University of Hamburg, Hamburg, Germany*
[a] *Corresponding author*
[†] *These authors contributed equally*
[*] *These authors jointly supervised the work*

Supplementary Note 1 BERT Robustness

BERT hierarchically applies the established algorithms ComBat [1] or limma [2] on a binary tree of pairwise batch-effect correction steps. The processing order of batches (i.e., the selection of batch pairs) is determined by their order in the user input. This section establishes the validity of this hierarchical approach both theoretically and experimentally, including a dedicated consideration of error propagation along the tree.

1.1 Mathematical Formalism

To help readers understand the mathematical justification for the hierarchical BERT approach, we provide a very short introduction to the frameworks used by BERT and refer the reader to the original publications and the respective source code for more detail.

1.1.1 limma

Let now $\mathbf{y}_i \in \mathbb{R}^m$ represent the raw (i.e., batch-effect afflicted) expression vector of feature i with one element for each of the m samples. Additionally, let $\mathbf{X} \in \mathbb{Z}^{m\times(p-1)}$ represent the deviation-coded design matrix containing the attribution of each sample to one of p batches. Limma then assumes the linear model

$$\mathbf{y}_i = \boldsymbol{\beta}_{0,i} + \mathbf{X}\boldsymbol{\beta}_i + \boldsymbol{\epsilon}_i, \quad \text{where} \quad \boldsymbol{\beta}_{0,i}, \boldsymbol{\epsilon}_i \in \mathbb{R}^m \quad \boldsymbol{\beta}_i \in \mathbb{R}^{p-1}, \tag{1}$$

$$= \begin{bmatrix} \mathbf{1} & \mathbf{X} \end{bmatrix} \begin{bmatrix} \beta_{0,i} \\ \boldsymbol{\beta}_i \end{bmatrix} + \boldsymbol{\epsilon}_i \tag{2}$$

$$\equiv \tilde{\mathbf{X}}\tilde{\boldsymbol{\beta}}_i + \boldsymbol{\epsilon}_i \tag{3}$$

Here, $\boldsymbol{\beta}_{0,i}$ denotes the vector with the average $\beta_{0,i}$ of mean expression values of i per batch in each component. The additive batch effects are modeled via $\boldsymbol{\beta}_i$ and can be estimated by minimizing the squared difference

$$\tilde{\boldsymbol{\beta}}_i^* = \arg\min_{\tilde{\boldsymbol{\beta}}_i} \left\| \mathbf{y}_i - \tilde{\mathbf{X}}\tilde{\boldsymbol{\beta}}_i \right\|_2 \tag{4}$$

between the experimental data and the theoretical model using ordinary least-squares regression. The algebraic solution is given by

$$\tilde{\boldsymbol{\beta}}_i^* = \left(\tilde{\mathbf{X}}^\top \tilde{\mathbf{X}}\right)^{-1} \tilde{\mathbf{X}}^\top \mathbf{y}_i, \tag{5}$$

and it follows for the batch-effect corrected data

$$\mathbf{y}_{i,corr.} = \mathbf{y}_i - \mathbf{X}\boldsymbol{\beta}_i^*. \tag{6}$$

As such, limma normalizes the mean expression values per batch to the respective global mean (i.e. $\beta_{0,i}$), and formulates this operation particularly efficiently using a least-squares regression. Note that the design matrix $\mathbf{X}$ can be modified to incorporate further contrasts (e.g., categorical covariates as considered in the main manuscript).

1.1.2 ComBat

The ComBat algorithm utilizes a generalized location and scale (L/S) model

$$\mathbf{y}_{ijk} = \boldsymbol{\alpha}_i + \mathbf{X}\boldsymbol{\beta}_i + \boldsymbol{\gamma}_{ik} + \boldsymbol{\delta}_{ik}\boldsymbol{\epsilon}_{ijk} \tag{7}$$

for the expression $\mathbf{y}_{ijk}$ of feature i, sample j and batch k. $\boldsymbol{\alpha}_i$ denotes the overall expression level per feature and $\boldsymbol{\gamma}_{ik}$, $\boldsymbol{\delta}_{ik}$ indicate additive and multiplicative batch-effect parameters, respectively.

In contrast to the limma method, the design matrix $\mathbf{X}$ now encodes biological or treatment groups (i.e., conditions to be preserved). Note that the noise $\boldsymbol{\epsilon}_{ijk} \sim \mathcal{N}(0, \boldsymbol{\sigma}_i^2)$ is multiplied to $\boldsymbol{\delta}_{ik}$ in an element-wise fashion.

To obtain unbiased Bayesian parameter estimates, the ComBat algorithm first computes standardized data

$$\mathbf{Z}_{ijk} = \frac{\mathbf{y}_{ijk} - \hat{\boldsymbol{\alpha}}_i - \mathbf{X}\hat{\boldsymbol{\beta}}_i}{\hat{\boldsymbol{\sigma}}_i}, \tag{8}$$

where feature-wise variances $\hat{\boldsymbol{\sigma}}_i$ across all features and samples are computed as

$$\hat{\boldsymbol{\sigma}}_i^2 = \frac{1}{m} \sum_{j,k} \left(\mathbf{y}_{ijk} - \hat{\boldsymbol{\alpha}}_i - \mathbf{X}\hat{\boldsymbol{\beta}}_i - \hat{\boldsymbol{\gamma}}_{ik} \right)^2 \tag{9}$$

based on constrained ordinary-least-squares estimates $\hat{\boldsymbol{\alpha}}_i$, $\hat{\boldsymbol{\beta}}_i$ and $\hat{\boldsymbol{\gamma}}_{ik}$.

For the Bayesian estimation, ComBat assumes that

$$\mathbf{Z}_{ijk} \sim \mathcal{N}\left(\boldsymbol{\gamma}_{ik}, \boldsymbol{\delta}_{ik}^2\right), \quad \text{where} \tag{10}$$

$$\boldsymbol{\gamma}_{ik} \sim \mathcal{N}\left(\boldsymbol{\gamma}_k, \boldsymbol{\tau}_k^2\right) \tag{11}$$

$$\boldsymbol{\delta}_{ik}^2 \sim \text{Inverse Gamma}\left(\boldsymbol{\lambda}_k, \boldsymbol{\Theta}_k\right). \tag{12}$$

Here, $\boldsymbol{\gamma}_k$, $\boldsymbol{\tau}_k^2$, $\boldsymbol{\lambda}_k$ and $\boldsymbol{\Theta}_k$ denote distributional hyperparameters, which can be estimated from the data matrix using straightforward operations. By employing Bayes theorem, the batch-effect parameters can then be estimated as

$$\boldsymbol{\gamma}_{ik}^* = \frac{s_k \bar{\boldsymbol{\tau}}_k^2 \hat{\gamma}_{ik} + \boldsymbol{\delta}_{ik}^{2*} \bar{\boldsymbol{\gamma}}_k}{s_k \bar{\boldsymbol{\tau}}_k^2 + \boldsymbol{\delta}_{ik}^{2*}} \tag{13}$$

$$\boldsymbol{\delta}_{ik}^{2*} = \frac{\bar{\boldsymbol{\Theta}}_k + \frac{1}{2} \sum_j^{s_k} \left(\mathbf{Z}_{ijk} - \boldsymbol{\gamma}_{ik}^*\right)^2}{\frac{s_k}{2} + \bar{\boldsymbol{\lambda}}_k - 1}, \tag{14}$$

where s_k denotes the number of samples in batch k and $\bar{\cdot}$ represent estimates of the distributional hyperparameters introduced above. The algorithm computes $\boldsymbol{\gamma}_{ik}^*$, $\boldsymbol{\delta}_{ik}^{2*}$ iteratively and stops computations if the estimates have converged (i.e., the absolute difference to the previous iteration is below a given threshold).

Finally, batch-effect corrected data can be computed as

$$\mathbf{y}_{ijk,corr.} = \hat{\boldsymbol{\epsilon}}_{ijk} + \hat{\boldsymbol{\alpha}}_i + \mathbf{X}\hat{\boldsymbol{\beta}}_i \tag{15}$$

$$= \frac{\mathbf{y}_{ijk} - \hat{\boldsymbol{\alpha}}_i - \mathbf{X}\hat{\boldsymbol{\beta}}_i - \hat{\boldsymbol{\gamma}}_{ik}}{\hat{\boldsymbol{\delta}}_{ik}} + \hat{\boldsymbol{\alpha}}_i + \mathbf{X}\hat{\boldsymbol{\beta}}_i \tag{16}$$

$$= \frac{\hat{\boldsymbol{\sigma}}_i}{\hat{\boldsymbol{\delta}}_{ik}} \left(\mathbf{Z}_{ijk} - \hat{\boldsymbol{\gamma}}_{ik}\right) + \hat{\boldsymbol{\alpha}}_i + \mathbf{X}\hat{\boldsymbol{\beta}}_i \tag{17}$$

$$\equiv \frac{\hat{\boldsymbol{\sigma}}_i}{\boldsymbol{\delta}_{ik}^*} \left(\mathbf{Z}_{ijk} - \boldsymbol{\gamma}_{ik}^*\right) + \hat{\boldsymbol{\alpha}}_i + \mathbf{X}\hat{\boldsymbol{\beta}}_i \tag{18}$$

As such, the ComBat algorithm uses the empirical Bayes method to estimate additive and multiplicative batch-effect parameters that allow to normalize each feature to the respective mean and to recover to batch-effect free noise term.

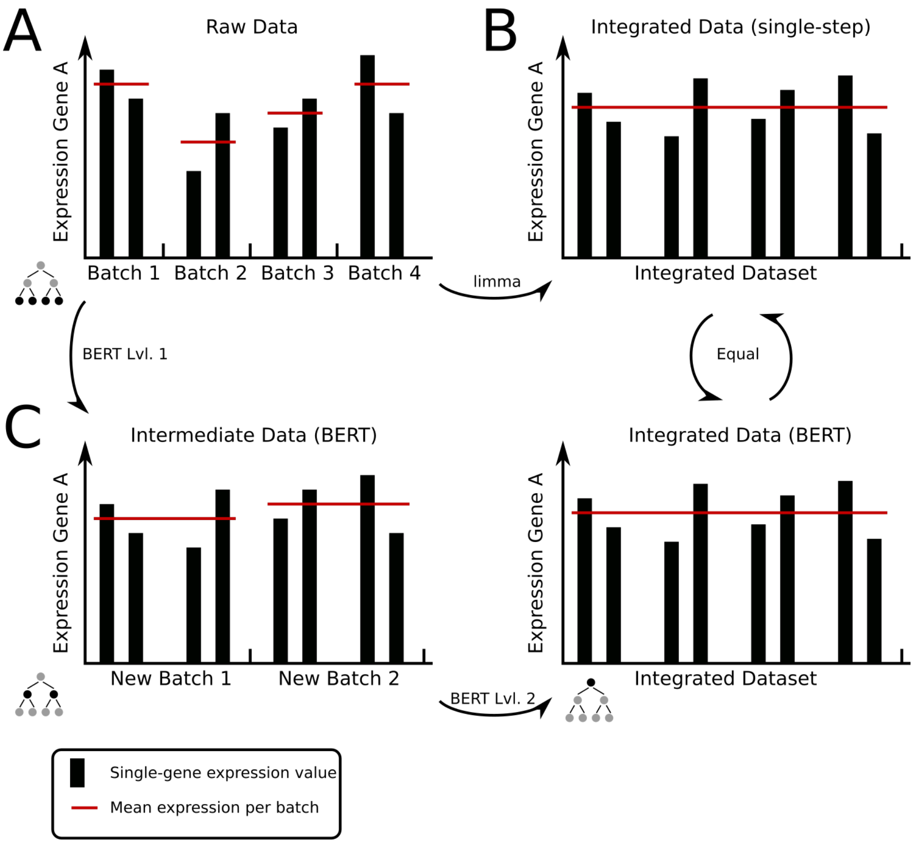

Supplementary Figure 1: *Concept sketch of single-step and hierarchical batch-effect correction with limma (four batches, two samples each).* **A** *Batch-effects in raw data lead to different mean expression per batch.* **B** *Integrating the batches in a single step aligns the mean expression values to each other.* **C** *With the hierarchical approach, intermediate batches are aggregated on BERT level 1 (bottom left), but the resulting data at the final BERT level 2 is identical to the single-step output (bottom right).*

1.1.3 BERT

Without loss of generality, we provide proof for the validity of the hierarchical approach of BERT for $4 = 2^2$ batches with the limma method, cf. Supplementary Fig. 1 A. Here, we consider complete data to compare the tree-based result of BERT to the results from a joint data integration of all four batches, where the latter would not be possible with missing data. All analytical expressions in this section were validated using the SymPy symbolic mathematics library [3] and the corresponding code has been made public in the accompanying code repository.

Let now each batch consist of two samples, where the expression vector of feature i follows the limma model (cf. Eq. (3)) and is given by

$$\mathbf{y} = \begin{pmatrix} \beta_0 + \beta_1 + \epsilon_1 \\ \beta_0 + \beta_1 + \epsilon_2 \\ \beta_0 + \beta_2 + \epsilon_3 \\ \beta_0 + \beta_2 + \epsilon_4 \\ \beta_0 + \beta_3 + \epsilon_5 \\ \beta_0 + \beta_3 + \epsilon_6 \\ \beta_0 + \beta_4 + \epsilon_7 \\ \beta_0 + \beta_4 + \epsilon_8 \end{pmatrix} \begin{matrix} \left.\begin{matrix} \\ \\ \end{matrix}\right\} \text{Batch } 1 \\ \left.\begin{matrix} \\ \\ \end{matrix}\right\} \text{Batch } 2 \\ \left.\begin{matrix} \\ \\ \end{matrix}\right\} \text{Batch } 3 \\ \left.\begin{matrix} \\ \\ \end{matrix}\right\} \text{Batch } 4 \end{matrix} . \tag{19}$$

Here, β_0 denotes the overall expression offset, β_1, ..., β_4 represent batch-specific biases and ϵ_1,...,ϵ_8 is the sample-wise Gaussian noise.

Single-step Correction The design matrix for the joint integration of all four batches reads

$$\tilde{\mathbf{X}} = \begin{pmatrix} 1 & 1 & 0 & 0 \\ 1 & 1 & 0 & 0 \\ 1 & 0 & 1 & 0 \\ 1 & 0 & 1 & 0 \\ 1 & 0 & 0 & 1 \\ 1 & 0 & 0 & 1 \\ 1 & -1 & -1 & -1 \\ 1 & -1 & -1 & -1 \end{pmatrix} \begin{matrix} \left.\begin{matrix} \\ \\ \end{matrix}\right\} \text{Batch 1} \\ \left.\begin{matrix} \\ \\ \end{matrix}\right\} \text{Batch 2} \\ \left.\begin{matrix} \\ \\ \end{matrix}\right\} \text{Batch 3} \\ \left.\begin{matrix} \\ \\ \end{matrix}\right\} \text{Batch 4} \end{matrix} , \tag{20}$$

where the different contrast scheme for batch four follows the rules for deviation coding. Then, the textbook-solution to the OLS regression problem (cf. Eq. (5)) yields

$$\left(\tilde{\mathbf{X}}^\top \tilde{\mathbf{X}}\right)^{-1} \tilde{\mathbf{X}}^\top = \begin{pmatrix} \frac{1}{8} & \frac{1}{8} & \frac{1}{8} & \frac{1}{8} & \frac{1}{8} & \frac{1}{8} & \frac{1}{8} & \frac{1}{8} \\ \frac{3}{8} & \frac{3}{8} & -\frac{1}{8} & -\frac{1}{8} & -\frac{1}{8} & -\frac{1}{8} & -\frac{1}{8} & -\frac{1}{8} \\ -\frac{1}{8} & -\frac{1}{8} & \frac{3}{8} & \frac{3}{8} & -\frac{1}{8} & -\frac{1}{8} & -\frac{1}{8} & -\frac{1}{8} \\ -\frac{1}{8} & -\frac{1}{8} & -\frac{1}{8} & -\frac{1}{8} & \frac{3}{8} & \frac{3}{8} & -\frac{1}{8} & -\frac{1}{8} \end{pmatrix} \tag{21}$$

$$\tilde{\boldsymbol{\beta}}^* = \begin{pmatrix} \beta_0 + \frac{\beta_1}{4} + \frac{\beta_2}{4} + \frac{\beta_3}{4} + \frac{\beta_4}{4} + \frac{\epsilon_1}{8} + \frac{\epsilon_2}{8} + \frac{\epsilon_3}{8} + \frac{\epsilon_4}{8} + \frac{\epsilon_5}{8} + \frac{\epsilon_6}{8} + \frac{\epsilon_7}{8} + \frac{\epsilon_8}{8} \\ \frac{3\beta_1}{4} - \frac{\beta_2}{4} - \frac{\beta_3}{4} - \frac{\beta_4}{4} + \frac{3\epsilon_1}{8} + \frac{3\epsilon_2}{8} - \frac{\epsilon_3}{8} - \frac{\epsilon_4}{8} - \frac{\epsilon_5}{8} - \frac{\epsilon_6}{8} - \frac{\epsilon_7}{8} - \frac{\epsilon_8}{8} \\ -\frac{\beta_1}{4} + \frac{3\beta_2}{4} - \frac{\beta_3}{4} - \frac{\beta_4}{4} - \frac{\epsilon_1}{8} - \frac{\epsilon_2}{8} + \frac{3\epsilon_3}{8} + \frac{3\epsilon_4}{8} - \frac{\epsilon_5}{8} - \frac{\epsilon_6}{8} - \frac{\epsilon_7}{8} - \frac{\epsilon_8}{8} \\ -\frac{\beta_1}{4} - \frac{\beta_2}{4} + \frac{3\beta_3}{4} - \frac{\beta_4}{4} - \frac{\epsilon_1}{8} - \frac{\epsilon_2}{8} - \frac{\epsilon_3}{8} - \frac{\epsilon_4}{8} + \frac{3\epsilon_5}{8} + \frac{3\epsilon_6}{8} - \frac{\epsilon_7}{8} - \frac{\epsilon_8}{8} \end{pmatrix} . \tag{22}$$

Thus, the results from the single-step integration of the four batches under consideration is given by

$$\mathbf{y}_{corr.,single-step} = \mathbf{y} - \mathbf{X}\boldsymbol{\beta}^* = \begin{pmatrix} \beta_0 + \frac{\beta_1}{4} + \frac{\beta_2}{4} + \frac{\beta_3}{4} + \frac{\beta_4}{4} + \frac{5\epsilon_1}{8} - \frac{3\epsilon_2}{8} + \frac{\epsilon_3}{8} + \frac{\epsilon_4}{8} + \frac{\epsilon_5}{8} + \frac{\epsilon_6}{8} + \frac{\epsilon_7}{8} + \frac{\epsilon_8}{8} \\ \beta_0 + \frac{\beta_1}{4} + \frac{\beta_2}{4} + \frac{\beta_3}{4} + \frac{\beta_4}{4} - \frac{3\epsilon_1}{8} + \frac{5\epsilon_2}{8} + \frac{\epsilon_3}{8} + \frac{\epsilon_4}{8} + \frac{\epsilon_5}{8} + \frac{\epsilon_6}{8} + \frac{\epsilon_7}{8} + \frac{\epsilon_8}{8} \\ \beta_0 + \frac{\beta_1}{4} + \frac{\beta_2}{4} + \frac{\beta_3}{4} + \frac{\beta_4}{4} + \frac{\epsilon_1}{8} + \frac{\epsilon_2}{8} + \frac{5\epsilon_3}{8} - \frac{3\epsilon_4}{8} + \frac{\epsilon_5}{8} + \frac{\epsilon_6}{8} + \frac{\epsilon_7}{8} + \frac{\epsilon_8}{8} \\ \beta_0 + \frac{\beta_1}{4} + \frac{\beta_2}{4} + \frac{\beta_3}{4} + \frac{\beta_4}{4} + \frac{\epsilon_1}{8} + \frac{\epsilon_2}{8} - \frac{3\epsilon_3}{8} + \frac{5\epsilon_4}{8} + \frac{\epsilon_5}{8} + \frac{\epsilon_6}{8} + \frac{\epsilon_7}{8} + \frac{\epsilon_8}{8} \\ \beta_0 + \frac{\beta_1}{4} + \frac{\beta_2}{4} + \frac{\beta_3}{4} + \frac{\beta_4}{4} + \frac{\epsilon_1}{8} + \frac{\epsilon_2}{8} + \frac{\epsilon_3}{8} + \frac{\epsilon_4}{8} + \frac{5\epsilon_5}{8} - \frac{3\epsilon_6}{8} + \frac{\epsilon_7}{8} + \frac{\epsilon_8}{8} \\ \beta_0 + \frac{\beta_1}{4} + \frac{\beta_2}{4} + \frac{\beta_3}{4} + \frac{\beta_4}{4} + \frac{\epsilon_1}{8} + \frac{\epsilon_2}{8} + \frac{\epsilon_3}{8} + \frac{\epsilon_4}{8} - \frac{3\epsilon_5}{8} + \frac{5\epsilon_6}{8} + \frac{\epsilon_7}{8} + \frac{\epsilon_8}{8} \\ \beta_0 + \frac{\beta_1}{4} + \frac{\beta_2}{4} + \frac{\beta_3}{4} + \frac{\beta_4}{4} + \frac{\epsilon_1}{8} + \frac{\epsilon_2}{8} + \frac{\epsilon_3}{8} + \frac{\epsilon_4}{8} + \frac{\epsilon_5}{8} + \frac{\epsilon_6}{8} + \frac{5\epsilon_7}{8} - \frac{3\epsilon_8}{8} \\ \beta_0 + \frac{\beta_1}{4} + \frac{\beta_2}{4} + \frac{\beta_3}{4} + \frac{\beta_4}{4} + \frac{\epsilon_1}{8} + \frac{\epsilon_2}{8} + \frac{\epsilon_3}{8} + \frac{\epsilon_4}{8} + \frac{\epsilon_5}{8} + \frac{\epsilon_6}{8} - \frac{3\epsilon_7}{8} + \frac{5\epsilon_8}{8} \end{pmatrix} . \tag{23}$$

Note that Eq. (23) consists of the constant offset β_0, the average of all batch-specific biases and a weighted sum of Gaussian noise terms that converges to zero for large number of samples.

Hierarchical Correction Following the hierarchical approach, BERT first integrates the batches 1,2 and 3,4 separately (first tree level), before integrating the resulting intermediate batches on the second tree level. The analytical solution to the OLS regression problem (cf. Eq. (5)) yields the coefficients

$$\tilde{\boldsymbol{\beta}}^*_{1+2} = \begin{pmatrix} \beta_0 + \frac{\beta_1}{2} + \frac{\beta_2}{2} + \frac{\epsilon_1}{4} + \frac{\epsilon_2}{4} + \frac{\epsilon_3}{4} + \frac{\epsilon_4}{4} \\ \frac{\beta_1}{2} - \frac{\beta_2}{2} + \frac{\epsilon_1}{4} + \frac{\epsilon_2}{4} - \frac{\epsilon_3}{4} - \frac{\epsilon_4}{4} \end{pmatrix} \tag{24}$$

$$\tilde{\boldsymbol{\beta}}^*_{3+4} = \begin{pmatrix} \beta_0 + \frac{\beta_3}{2} + \frac{\beta_4}{2} + \frac{\epsilon_5}{4} + \frac{\epsilon_6}{4} + \frac{\epsilon_7}{4} + \frac{\epsilon_8}{4} \\ \frac{\beta_3}{2} - \frac{\beta_4}{2} + \frac{\epsilon_5}{4} + \frac{\epsilon_6}{4} - \frac{\epsilon_7}{4} - \frac{\epsilon_8}{4} \end{pmatrix} \tag{25}$$

We refer the reader to the corresponding Eq. (22) of the single-step approach for comparison. The respective intermediate batches

$$\mathbf{y}_{1+2,corr.} = \begin{pmatrix} \beta_0 + \frac{\beta_1}{2} + \frac{\beta_2}{2} + \frac{3\epsilon_1}{4} - \frac{\epsilon_2}{4} + \frac{\epsilon_3}{4} + \frac{\epsilon_4}{4} \\ \beta_0 + \frac{\beta_1}{2} + \frac{\beta_2}{2} - \frac{\epsilon_1}{4} + \frac{3\epsilon_2}{4} + \frac{\epsilon_3}{4} + \frac{\epsilon_4}{4} \\ \beta_0 + \frac{\beta_1}{2} + \frac{\beta_2}{2} + \frac{\epsilon_1}{4} + \frac{\epsilon_2}{4} + \frac{3\epsilon_3}{4} - \frac{\epsilon_4}{4} \\ \beta_0 + \frac{\beta_1}{2} + \frac{\beta_2}{2} + \frac{\epsilon_1}{4} + \frac{\epsilon_2}{4} - \frac{\epsilon_3}{4} + \frac{3\epsilon_4}{4} \end{pmatrix} \tag{26}$$

$$\mathbf{y}_{3+4,corr.} = \begin{pmatrix} \beta_0 + \frac{\beta_3}{2} + \frac{\beta_4}{2} + \frac{3\epsilon_5}{4} - \frac{\epsilon_6}{4} + \frac{\epsilon_7}{4} + \frac{\epsilon_8}{4} \\ \beta_0 + \frac{\beta_3}{2} + \frac{\beta_4}{2} - \frac{\epsilon_5}{4} + \frac{3\epsilon_6}{4} + \frac{\epsilon_7}{4} + \frac{\epsilon_8}{4} \\ \beta_0 + \frac{\beta_3}{2} + \frac{\beta_4}{2} + \frac{\epsilon_5}{4} + \frac{\epsilon_6}{4} + \frac{3\epsilon_7}{4} - \frac{\epsilon_8}{4} \\ \beta_0 + \frac{\beta_3}{2} + \frac{\beta_4}{2} + \frac{\epsilon_5}{4} + \frac{\epsilon_6}{4} - \frac{\epsilon_7}{4} + \frac{3\epsilon_8}{4} \end{pmatrix} \tag{27}$$

are then integrated by means of

$$\tilde{\mathbf{X}}_{\substack{(1+2)\\(3+4)}} = \begin{pmatrix} 1 & 1 \\ 1 & 1 \\ 1 & 1 \\ 1 & 1 \\ 1 & -1 \\ 1 & -1 \\ 1 & -1 \\ 1 & -1 \end{pmatrix} \tag{28}$$

$$\left(\tilde{\mathbf{X}}^\top_{\substack{(1+2)\\(3+4)}} \tilde{\mathbf{X}}_{\substack{(1+2)\\(3+4)}} \right)^{-1} \tilde{\mathbf{X}}^\top_{\substack{(1+2)\\(3+4)}} = \begin{pmatrix} \frac{1}{8} & \frac{1}{8} & \frac{1}{8} & \frac{1}{8} & \frac{1}{8} & \frac{1}{8} & \frac{1}{8} & \frac{1}{8} \\ \frac{1}{8} & \frac{1}{8} & \frac{1}{8} & \frac{1}{8} & -\frac{1}{8} & -\frac{1}{8} & -\frac{1}{8} & -\frac{1}{8} \end{pmatrix} \tag{29}$$

$$\tilde{\boldsymbol{\beta}}^*_{\substack{(1+2)\\(3+4)}} = \begin{pmatrix} \beta_0 + \frac{\beta_1}{4} + \frac{\beta_2}{4} + \frac{\beta_3}{4} + \frac{\beta_4}{4} + \frac{\epsilon_1}{8} + \frac{\epsilon_2}{8} + \frac{\epsilon_3}{8} + \frac{\epsilon_4}{8} + \frac{\epsilon_5}{8} + \frac{\epsilon_6}{8} + \frac{\epsilon_7}{8} + \frac{\epsilon_8}{8} \\ \frac{\beta_1}{4} + \frac{\beta_2}{4} - \frac{\beta_3}{4} - \frac{\beta_4}{4} + \frac{\epsilon_1}{8} + \frac{\epsilon_2}{8} + \frac{\epsilon_3}{8} + \frac{\epsilon_4}{8} - \frac{\epsilon_5}{8} - \frac{\epsilon_6}{8} - \frac{\epsilon_7}{8} - \frac{\epsilon_8}{8} \end{pmatrix}, \tag{30}$$

yielding

$$\mathbf{y}_{\substack{(1+2)\\(3+4)},corr.} = \begin{pmatrix} \beta_0 + \frac{\beta_1}{4} + \frac{\beta_2}{4} + \frac{\beta_3}{4} + \frac{\beta_4}{4} + \frac{5\epsilon_1}{8} - \frac{3\epsilon_2}{8} + \frac{\epsilon_3}{8} + \frac{\epsilon_4}{8} + \frac{\epsilon_5}{8} + \frac{\epsilon_6}{8} + \frac{\epsilon_7}{8} + \frac{\epsilon_8}{8} \\ \beta_0 + \frac{\beta_1}{4} + \frac{\beta_2}{4} + \frac{\beta_3}{4} + \frac{\beta_4}{4} - \frac{3\epsilon_1}{8} + \frac{5\epsilon_2}{8} + \frac{\epsilon_3}{8} + \frac{\epsilon_4}{8} + \frac{\epsilon_5}{8} + \frac{\epsilon_6}{8} + \frac{\epsilon_7}{8} + \frac{\epsilon_8}{8} \\ \beta_0 + \frac{\beta_1}{4} + \frac{\beta_2}{4} + \frac{\beta_3}{4} + \frac{\beta_4}{4} + \frac{\epsilon_1}{8} + \frac{\epsilon_2}{8} + \frac{5\epsilon_3}{8} - \frac{3\epsilon_4}{8} + \frac{\epsilon_5}{8} + \frac{\epsilon_6}{8} + \frac{\epsilon_7}{8} + \frac{\epsilon_8}{8} \\ \beta_0 + \frac{\beta_1}{4} + \frac{\beta_2}{4} + \frac{\beta_3}{4} + \frac{\beta_4}{4} + \frac{\epsilon_1}{8} + \frac{\epsilon_2}{8} - \frac{3\epsilon_3}{8} + \frac{5\epsilon_4}{8} + \frac{\epsilon_5}{8} + \frac{\epsilon_6}{8} + \frac{\epsilon_7}{8} + \frac{\epsilon_8}{8} \\ \beta_0 + \frac{\beta_1}{4} + \frac{\beta_2}{4} + \frac{\beta_3}{4} + \frac{\beta_4}{4} + \frac{\epsilon_1}{8} + \frac{\epsilon_2}{8} + \frac{\epsilon_3}{8} + \frac{\epsilon_4}{8} + \frac{5\epsilon_5}{8} - \frac{3\epsilon_6}{8} + \frac{\epsilon_7}{8} + \frac{\epsilon_8}{8} \\ \beta_0 + \frac{\beta_1}{4} + \frac{\beta_2}{4} + \frac{\beta_3}{4} + \frac{\beta_4}{4} + \frac{\epsilon_1}{8} + \frac{\epsilon_2}{8} + \frac{\epsilon_3}{8} + \frac{\epsilon_4}{8} - \frac{3\epsilon_5}{8} + \frac{5\epsilon_6}{8} + \frac{\epsilon_7}{8} + \frac{\epsilon_8}{8} \\ \beta_0 + \frac{\beta_1}{4} + \frac{\beta_2}{4} + \frac{\beta_3}{4} + \frac{\beta_4}{4} + \frac{\epsilon_1}{8} + \frac{\epsilon_2}{8} + \frac{\epsilon_3}{8} + \frac{\epsilon_4}{8} + \frac{\epsilon_5}{8} + \frac{\epsilon_6}{8} + \frac{5\epsilon_7}{8} - \frac{3\epsilon_8}{8} \\ \beta_0 + \frac{\beta_1}{4} + \frac{\beta_2}{4} + \frac{\beta_3}{4} + \frac{\beta_4}{4} + \frac{\epsilon_1}{8} + \frac{\epsilon_2}{8} + \frac{\epsilon_3}{8} + \frac{\epsilon_4}{8} + \frac{\epsilon_5}{8} + \frac{\epsilon_6}{8} - \frac{3\epsilon_7}{8} + \frac{5\epsilon_8}{8} \end{pmatrix}. \tag{31}$$

Note that $\mathbf{y}_{\substack{(1+2)\\(3+4)},corr.}$ is identical to $\mathbf{y}_{corr.,single-step}$, cf. Supplementary Fig 1 B,C. We argue that any tree with $N = 2^m$, $m \in \mathbb{N}$ batches can be decomposed into nested sub-trees with four batches each and that the demonstrated validity of the hierarchical approach hence extends to larger trees as well. Indeed, exemplary simulated data with 32 batches and 10000 features confirmed this relation with a mean absolute difference of $\mathcal{O}(10^{-15})$ between the hierarchical result from BERT and a single correction step with limma. Importantly, additional symbolic analyses with Sympy showed that the above findings are independent of the number of samples per batch (tested 50 repetitions with random numbers $n \in [2, 50]$ of samples per batch). Note further that both $\mathbf{y}_{\substack{(1+2)\\(3+4)},corr.}$ and $\mathbf{y}_{corr.,single-step}$ are invariant under batch input order (e.g., try substituting β_1, ϵ_1, ϵ_2 by β_2, ϵ_3, ϵ_4 and vice versa). Again, this behaviour must hold for any sub-tree that can be decomposed into

nested sub-trees with four input batches each.

For odd number of batches, or if a subset of numerical values is propagated to the next tree level without batch-effect correction since the other batch did not contain matching numerical values, a permutation of batches may introduce an additive offset on each feature (constant across all batches + Gaussian Noise). As an example, consider the first three batches of the data integration problem considered above (Eq. (19)). Integrating batches one and two, followed by an integration with batch three on the second tree level yields

$$\mathbf{y}_{\substack{(1+2)\\(3)},corr.} = \begin{pmatrix} \beta_0 + \frac{\beta_1}{4} + \frac{\beta_2}{4} + \frac{\beta_3}{2} + \frac{5\epsilon_1}{8} - \frac{3\epsilon_2}{8} + \frac{\epsilon_3}{8} + \frac{\epsilon_4}{8} + \frac{\epsilon_5}{2} \\ \beta_0 + \frac{\beta_1}{4} + \frac{\beta_2}{4} + \frac{\beta_3}{2} - \frac{3\epsilon_1}{8} + \frac{5\epsilon_2}{8} + \frac{\epsilon_3}{8} + \frac{\epsilon_4}{8} + \frac{\epsilon_5}{2} \\ \beta_0 + \frac{\beta_1}{4} + \frac{\beta_2}{4} + \frac{\beta_3}{2} + \frac{\epsilon_1}{8} + \frac{\epsilon_2}{8} + \frac{5\epsilon_3}{8} - \frac{3\epsilon_4}{8} + \frac{\epsilon_5}{2} \\ \beta_0 + \frac{\beta_1}{4} + \frac{\beta_2}{4} + \frac{\beta_3}{2} + \frac{\epsilon_1}{8} + \frac{\epsilon_2}{8} - \frac{3\epsilon_3}{8} + \frac{5\epsilon_4}{8} + \frac{\epsilon_5}{2} \\ \beta_0 + \frac{\beta_1}{4} + \frac{\beta_2}{4} + \frac{\beta_3}{2} + \frac{\epsilon_1}{8} + \frac{\epsilon_2}{8} + \frac{\epsilon_3}{8} + \frac{\epsilon_4}{8} + \frac{\epsilon_5}{2} \\ \beta_0 + \frac{\beta_1}{4} + \frac{\beta_2}{4} + \frac{\beta_3}{2} + \frac{\epsilon_1}{8} + \frac{\epsilon_2}{8} + \frac{\epsilon_3}{8} + \frac{\epsilon_4}{8} + \frac{\epsilon_5}{2} \end{pmatrix}, \tag{32}$$

whereas the initial integration of batch two, three followed by an integration with batch one yields

$$\mathbf{y}_{\substack{(2+3)\\(1)},corr.} = \begin{pmatrix} \beta_0 + \frac{\beta_1}{2} + \frac{\beta_2}{4} + \frac{\beta_3}{4} + \frac{\epsilon_1}{2} + \frac{\epsilon_3}{8} + \frac{\epsilon_4}{8} + \frac{\epsilon_5}{8} + \frac{\epsilon_6}{8} \\ \beta_0 + \frac{\beta_1}{2} + \frac{\beta_2}{4} + \frac{\beta_3}{4} + \frac{\epsilon_1}{2} + \frac{\epsilon_3}{8} + \frac{\epsilon_4}{8} + \frac{\epsilon_5}{8} + \frac{\epsilon_6}{8} \\ \beta_0 + \frac{\beta_1}{2} + \frac{\beta_2}{4} + \frac{\beta_3}{4} + \frac{\epsilon_1}{2} + \frac{5\epsilon_3}{8} - \frac{3\epsilon_4}{8} + \frac{\epsilon_5}{8} + \frac{\epsilon_6}{8} \\ \beta_0 + \frac{\beta_1}{2} + \frac{\beta_2}{4} + \frac{\beta_3}{4} + \frac{\epsilon_1}{2} - \frac{3\epsilon_3}{8} + \frac{5\epsilon_4}{8} + \frac{\epsilon_5}{8} + \frac{\epsilon_6}{8} \\ \beta_0 + \frac{\beta_1}{2} + \frac{\beta_2}{4} + \frac{\beta_3}{4} + \frac{\epsilon_1}{2} + \frac{\epsilon_3}{8} + \frac{\epsilon_4}{8} + \frac{5\epsilon_5}{8} - \frac{3\epsilon_6}{8} \\ \beta_0 + \frac{\beta_1}{2} + \frac{\beta_2}{4} + \frac{\beta_3}{4} + \frac{\epsilon_1}{2} + \frac{\epsilon_3}{8} + \frac{\epsilon_4}{8} - \frac{3\epsilon_5}{8} + \frac{5\epsilon_6}{8} \end{pmatrix}. \tag{33}$$

The element-wise difference between the integrated data corresponds to

$$-\frac{\beta_1}{4} + \frac{\beta_3}{4} + \underbrace{\text{Weighted Mean of } \epsilon_1, \cdot, \epsilon_6}_{\approx 0} \tag{34}$$

for all samples, such that $\mathbf{y}_{\substack{(1+2)\\(3)},corr.}$, $\mathbf{y}_{\substack{(2+3)\\(1)},corr.}$. This additive constant offset can be understood intuitively by looking at Supplementary Fig. 1 – the limma model effectively shifts the mean of each considered batch to their respective grand average (at each node of the respective tree level). If a batch is only considered at a later tree level, this results in a different grand mean at the final node – hence the constant offset of the result.
Please note that such offset vanishes under the common z-score normalization procedure and that the weighted mean of Gaussian noise approaches the zero rapidly with increasing number of samples per batch. We argue, that the offset in Eq. (34) is irrelevant for the anticipated workflows and downstream tasks of users including differential expression analysis, classification and others, which exclusively rely on relative differences of expression values between samples. Moreover, users will typically apply domain-specific normalization procedures to each feature after batch-effect correction, such as sample-wise z-score normalization, which diminishes the introduced offset.

Instead of the simple OLS method limma, the ComBat algorithm uses the empirical Bayes method to infer additive and multiplicate batch-effects and removes them from a more general L/S-model, cf. Eq. 7. The corrected data is computed using parameters $\hat{\boldsymbol{\alpha}}_i$, $\hat{\boldsymbol{\beta}}_i$, $\hat{\boldsymbol{\gamma}}_i$ from robust, constrained least-squares optimization and an estimate of the biological/technical variation computed using the Bayes estimates $\boldsymbol{\delta}^*_{ik}$, $\boldsymbol{\gamma}^*_{ik}$. While a similar theoretical analysis as above would be feasible for the least-squares parameters, such a statement can not be established easily for the Bayes parameters due to the heuristic stopping criterion of the parameter estimation method. We hence provide additional experimental error estimation for the hierarchical BERT approach with ComBat, to complement the verification on *real* data in the main manuscript by means of simulated datasets.

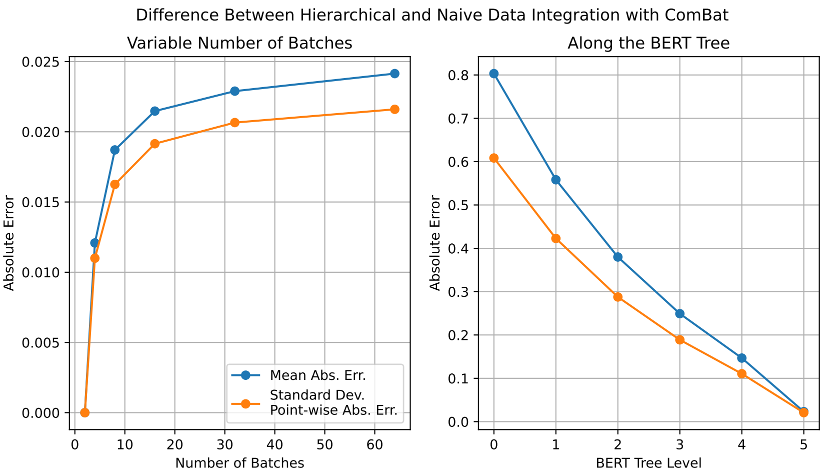

Supplementary Figure 2: *Mean absolute error between hierarchical (BERT) and naïve data integration with ComBat, as well as the respective point-wise standard deviation. Left: The error and standard deviation increase with the number of input batches (2-64), but the error flattens after 8 batches. Right: The error and standard deviation decrease continously with each tree level (0 – leaf level, 5 – integrated data). Source data are provided as a Source Data file.*

To this end, we first considered simulated complete data with 32 batches, 10.000 features, 10 samples per batch and 2 simulated biological conditions, and confirmed for limma that the hierarchical BERT-approach yielded identical results as a single-step correction (almost to machine precision, mean absolute error of $3.0 \cdot 10^{-15}$ or better in 10 independent simulations, maximum point-wise error $< 3.8 \cdot 10^{-14}$). For ComBat, we found the mean absolute error between hierarchical and naïve data integration, as well as the respective standard deviation of absolute point-wise errors, to increase with the number of batches cf. left panel of Supplementary Fig. 2 (only very small increase after 8 batches). Here, the iterative solver of ComBat was manually configured for a particularly high precision of 10^{-10}%.
Furthermore, we performed additional parameter studies, randomly sampling 50 independent datasets with batches in $[2, 4, 8, \cdots, 64]$, feature counts in $[2000, 4000, \cdots, 18000, 20000]$, and samples per batch in $[5, 10, 15, 20]$. The maximum mean absolute error observed across all drawn configurations was < 0.042, which is well below the recommended fold-change (FC) cutoffs (e.g., $\log_2$ FC ≥ 1, cf. [4]) as well as below the first moment of absolute expression differences between the simulated biological conditions (here: $\sqrt{\frac{2}{\pi}}$). Note that the maximum point-wise error observed across all parameter combinations and any protein/batch/sample varied strongly (observations in $[9 \cdot 10^{-16}, 0.621]$), but remained well below these thresholds required in practice.
Finally, we found the mean absolute error as well as the point-wise standard deviations to decrease continuously along the BERT tree levels, indicating stable convergence (cf. right panel of Supplementary Fig. 2). In summary, these findings support the methodological validity of the hierarchical BERT approach on such simulated data by confirming that the hierarchical approach yields comparable results to the naïve data integration with ComBat for various data sizes. The reader is referred to the main manuscript for further experimental validation on *real omic* data.

1.1.4 Experimental Validation

In addition to the above experimental validation for ComBat, we now investigate whether any errors from early tree levels could be propagated to later tree levels and hence distort the batch-effect corrected data. We now assume that any such error would be specific to the respectively adjusted pair of batches and conclude that the distortion of the BERT output would then depend on the order

Supplementary Table 1: *Mean and standard deviation of the difference between ASW scores, as well as the median absolute difference (MAD) of z-score normalized expression values for two permutations of 50 independently drawn datasets each.*

	**ASW Batch**	**ASW Label**	**MAD**
**ComBat**	$4.94E{-}04 \pm 3.95E{-}04$	$3.61E{-}04 \pm 2.88E{-}04$	$1.47E{-}02 \pm 1.51E{-}03$
**limma**	$2.11E{-}17 \pm 1.85E{-}17$	$8.88E{-}18 \pm 3.01E{-}17$	$1.56E{-}15 \pm 2.18E{-}17$

of batches in the input as well. We therefore investigate and quantify the robustness and the effect of error propagation by measuring the variability of BERT output for different batch permutations.

Using two permutations each, the absolute difference of ASW scores and median absolute difference (MAD) of z-score normalized expression values was determined on 50 simulated datasets with 6000 features and 30% missing values (30 batches, 10 samples per batch), cf. Supplementary Tab. 1. For limma, the observed mean variation was close to machine precision ($\mathcal{O}(10^{-17})$ and $\mathcal{O}(10^{-15})$ for ASW scores and expression differences, respectively), indicating the absence of any accumulated batch-specific errors. For ComBat, variation was higher ($\mathcal{O}(10^{-4})$ and $\mathcal{O}(10^{-2})$, respectively) but remained significantly below typical effects users observe for biological or technical reasons.

With respect to the theoretical analysis of robustness in Sec. 1.1.3, these results confirm the robustness of the hierarchical BERT method with respect to batch order – and hence also confirm that it does not incur error accumulation across tree levels. For ComBat in particular, we suspect that the increased variability can be attributed to the iterative procedure and heuristic stopping criterion of the algorithm [1]. Yet, the observed variability for both algorithms remains well below typical variability observed by users in practice e.g., from biological variation.

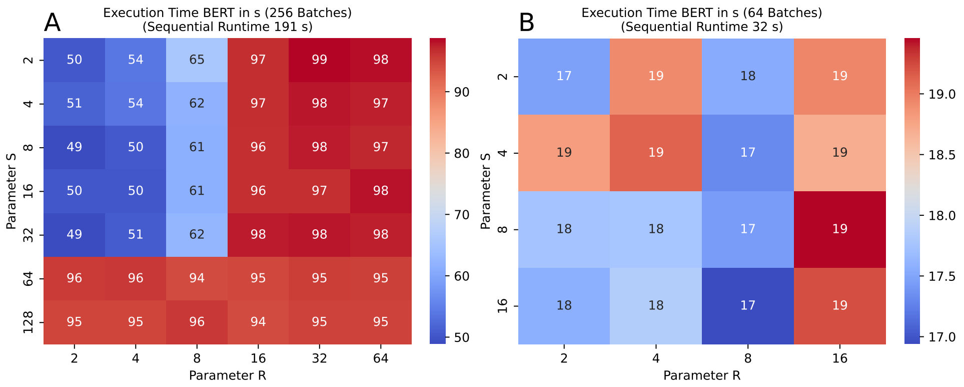

Supplementary Figure 3: *Heatmap with average execution time for batch-effect correction with BERT using various values for the parameters R and S (five repetitions, 64 processes (left) and 16 processes (right)). Sequential execution time is indicated in the respective heading of each panel. Source data are provided as a Source Data file.*

Supplementary Note 2 **BERT Parameters for Parallelization**

Due to the tree structure of the BERT algorithm, integration steps from different branches are strictly independent. Therefore, the algorithm may be executed in parallel or sequentially without any impact on the result. In addition to the number of processes P (typically limited by the user hardware) and the inter-process communication backend (e.g., selecting de-/serialization from temporary filesystem may be faster on specific, non-commodity hardware), the BERT parallelization scheme is controlled via user-defined parameters R (factor to reduce number of processes after subtree-correction) and S (number of remaining batches to stop further parallelization). While the BERT-library provides reasonable defaults for typical commodity-hardware ($R = 4$, $S = 2$), users may wish to adapt these defaults to their problem sizes and computer.

Supplementary Fig. 3 reports average execution times for BERT correction with various R, S for the two strong scaling experiments from the main manuscript (64 and 16 processes, respectively). Here, each batch comprises 10 samples, drawn from two simulated conditions, with 6000 features and 10% missing values each. It is apparent that R, S influence execution time considerably (e.g., optimum of 49s compared to to maximum of 99s). Based on the reported experiments, users should test low R and S (e.g. 2, 4) to avoid preemptive reduction of processes or even termination of parallel execution.

With increasing batch sizes (and hence communication costs), we expect the optimal R, S, to increase slightly. Note that the parameters R, S, as well as the number of processes P, affect only the execution time and do not change the BERT-output.

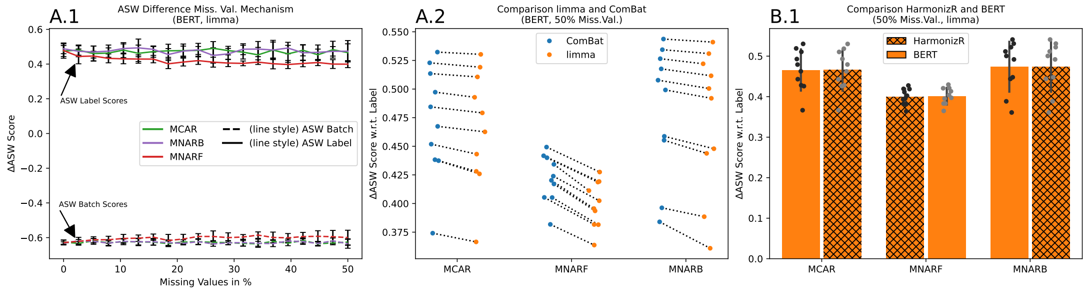

Supplementary Figure 4: *Results from simulation studies on the effect of missing value mechanisms on the output of BERT.* **A** *Mean and standard deviation of ASW score differences between input and BERT's output for variable amount of missing values (limma, 10 repetitions) (A.1) and ASW label score differences for BERT correction using either ComBat or limma (A.2, 50% missing values).* **B.1** *Mean and standard deviation of ASW label for BERT and HarmonizR (10 repetitions). Source data are provided as a Source Data file.*

Supplementary Note 3 **Robustness to Missing Value Types**

The data generating process described in the main manuscript of this study was explicitly designed to mimic the typical distribution of missing values for mass-spectrometric measurements (i.e., full completeness or absence of a feature per batch). Following the common distinction of missing values into *MCAR* (missing completely at random), *MAR* (missing at random) and *MNAR* (missing not at random) based on formal statistical properties [5, 6], this scheme represents a feature-wise MCAR mechanism. In practice, detection limits of the employed measurement devices may introduce left-censored data (i.e., MNAR data), especially for metabolomics [7]. To assert validity of BERT under such conditions, we conducted additional simulation studies to provide a formal characterization of the algorithm for MCAR data.

Simulated data with 6000 features, 20 batches with 10 samples each and two biological conditions was generated using the procedure described in the *Online Methods* (10 independently generated datasets). A varying ratio of up to 50% missing values was inserted by

(1) selecting a random subset of features to be missing completely for the entire batch (MCAR, as in main manuscript)

(2) selecting the lowest simulated expression values (*features*) of the entire data matrix to be missing (referred to as MNARF in the following, similar to dropout from detection limits e.g., in mass-spetrometry based metabolomics)

(3) selecting the appropriate number of features with lowest mean expression value per *batch* to be missing completely for the entire batch (referred to as MNARB, e.g. representing dropout in typical DDA acquisition in mass-spectrometric proteomics with isobaric labels).

ASW scores were computed for the raw, as well as the corrected data from BERT and HarmonizR, respectively (both with limma and ComBat).

The mean difference of ASW scores of the raw and batch-effect corrected data remained approximately constant for the considered amounts of missing data (e.g., max. abs. diff. between mean ASW label differences of 0.02, 0.02 and 0.04 for MCAR, MNARB and MNARF, respectively using BERT with limma, cf. Supplementary Fig. 4 A.1). No significant difference of the mean ASW improvements could be observed for MCAR and MNARB (paired t-test, $p > 0.8$), whereas the difference for MCAR and MNARF was significant ($p < 4 \cdot 10^{-9}$) but the effect size was limited (Cohen's $d \approx 0.79$). Higher improvements of ASW label scores were observed using ComBat instead of limma (e.g., mean difference of 0.01, 0.02 and 0.01 for MCAR, MNARF and MNARB, respectively, at 50% missing values, cf. Supplementary Fig. 4 A.2). Of note, no difference was observed between Harmo-

nizR and BERT for all three missing value mechanisms (e.g., $p > 0.27$ for MCAR, cf. Supplementary Fig. 4 B.1).

From the above analyses, we conclude that the MCAR-like simulation scheme in the main manuscript is sufficiently representative for BERT's and HarmonizR's behaviour over the considered range of missing values. Furthermore, the results confirm explicitly that BERT is suitable for MNAR data, as found for example in metabolomics, which emphasizes the broad scope of the algorithm. Experiments on real-world metabolomic data in the main manuscript support this interpretation.

Supplementary Table 2: *Mean accuracy and respective standard deviation of a k-nearest neighbor classifier on raw and batch-effect corrected data (10 repetitions). Considerably reduced accuracy was observed for the raw input (grey background).*

	BERT		HarmonizR		
**Missing Values**	**ComBat**	**limma**	**ComBat**	**limma**	**raw**
**0.0 %**	1.0 ± 0.0	1.0 ± 0.0	1.0 ± 0.0	1.0 ± 0.0	0.821 ± 0.087
**18.42 %**	1.0 ± 0.0	1.0 ± 0.0	1.0 ± 0.0	1.0 ± 0.0	0.879 ± 0.067
**26.32 %**	1.0 ± 0.0	1.0 ± 0.0	1.0 ± 0.0	1.0 ± 0.0	0.888 ± 0.049
**36.84 %**	1.0 ± 0.0	1.0 ± 0.0	1.0 ± 0.0	1.0 ± 0.0	0.882 ± 0.068
**47.37 %**	1.0 ± 0.0	1.0 ± 0.0	1.0 ± 0.0	1.0 ± 0.0	0.91 ± 0.058

Supplementary Note 4 **Improvement of Classification by Batch-Effect Correction**

Across various *omic* domains, batch-effects represent a prominent limitation for the application of machine-learning methods, as highlighted by several authors [8–11]. In particular, *classification* represents a clinically highly relevant tasks e.g., distinguishing benign or malignant neoplasms. In addition to the evaluation on *omic* data in Sec. 2 of the main manuscript, we hence also evaluate the capability of BERT and HarmonizR to improve the performance of a typical classification algorithm on simulated data.

Simulated data with 6000 features, 20 batches (10 samples per batch), 10 simulated biological conditions and a variable amount of missing values between 0% and approx. 50% was generated in 10 repetitions as described in the Online Methods. For both the raw and the batch-effect corrected data (BERT and HarmonizR with limma and ComBat each), the accuracy of a k-nearest neighbor (kNN) classifier was determined on a randomly chosen 25% test split ($k = 1$) of each generated dataset. Here, Euclidean distance matrices were used for classification, as to avoid imputation on the highly incomplete data. While the average accuracy of the kNN-classifier was significantly reduced on the raw data (i.e., including the simulated batch effects), BERT and HarmonizR yielded batch-effect corrected data with flawless classification results for both ComBat and limma (cf. Supplementary Tab. 2).

For this experiment, the model parameters to generate the simulated data have been left to their defaults to maintain consistency with the main manuscript. Importantly, the results obtained here confirm the results obtained using the ASW scores, where significantly improved and approximately constant scores were reported for BERT in Fig. 3 (B.1 and B.2). In summary, the experiment demonstrates that batch-effect correction (e.g., with BERT) represents a key step in data preprocessing for classification tasks. The reader is referred to Sec. 2 of the main manuscript for an example on real-world *omic* data.

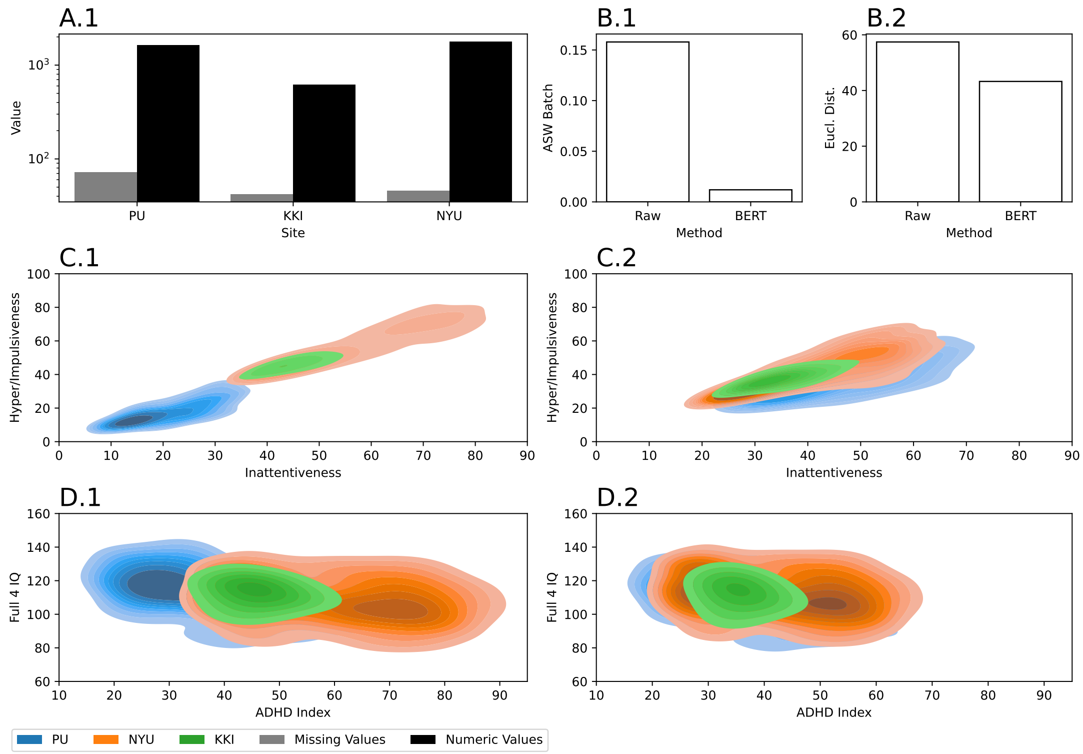

Supplementary Figure 5: *Results for BERT applied to phenotypic data from the ADHD200 cohort. Outer bounds of kernel density estimates exclude the outermost 20% of samples.* **A.1** *Absolute number of numeric and missing values per site (Peking University/PU, Kennedy Krieger Institute/KKI, New York University Langone Medical Center/NYU).* **B** *Average silhouette score (ASW) with respect to batch of origin (***B.1***) and average pairwise Euclidean distance (***B.2***) between the samples before and after batch-effect correction with BERT (ComBat, non-parametric priors).* **C** *Kernel-density estimates of inattentiveness and hyper/impulsiveness per partner site on the raw (***C.1***) and BERT-corrected data (***C.2***).* **D** *Kernel-density estimates of ADHD Index and Full 4 IQ per partner site on the raw (***D.1***) and BERT-corrected data (***D.2***).*

Supplementary Note 5 **Application of BERT to the ADHD200 Dataset**

Preprocessed phenotypic information of the ADHD200 cohort (*Consolidated ADHD200 phenotypics version 1.0*) was obtained from the Neuro Bureau via the Neuroimaging Informatics Tools and Resources Clearinghouse (NITRC) [12]. Samples were selected from the *Peking University* (PU), the *Kennedy Krieger Institute* (KKI) and the *New York University Langone Medical Center* (NYU). Considerations were restricted to the Attention-Deficit Hyperactivity Disorder (ADHD) index, the inattentiveness, the hyper/impulsiveness, the verbal IQ, the performance IQ and the full 4 IQ. It has been shown in a statistical analysis [13] of the ADHD200 cohort, that inattention was conditionally independent of sex distribution and any difference between the sites of origin was hence defined as batch-effect in the following proof-of-concept experiment. In practice, researchers need to verify such an assumption for their dataset or account for imbalanced study design by means of covariates or references.

The respective number of numeric and missing values per site are reported in Fig. 5 A.1. The data was corrected for batch-effects with respect to the medical centers using ComBat with non-parametric priors. It was observed that both the ASW with respect to batch (i.e., site of origin) as well as the average Euclidean distance between all samples decreased considerably compared to the raw data, cf. Fig. 5 B.1 and B.2, respectively. In the raw data, the distributions of inattentiveness and hyper/impulsiveness differed strongly between the sites, cf. Fig. 5 C.1. After BERT, the respective distributions were more similar and exhibited lower standard deviation, which in summary indicated

successful batch-effect correction, cf. Fig. 5 C.2. Similarly, distributional similarity and sample variability of the ADHD index and the Full 4 IQ was improved with BERT (cf. Fig. 5 D.1 and D.2) for raw and processed data, respectively).

Supplementary Note 6 **BERT Scaling**

In order to rigorously justify the suitability of BERT for datasets with large number of batches, we derive the theoretical scaling behaviour of BERT from first principles. Let now $N = 2^M$ denote the number of batches and M represent the number of BERT tree levels.

Linear Correction Time Let the number of observations per batch be constant across all batches and let $2C$ denote the respective batch-effect correction time per pair of batches at leaf level ($C \in \mathbb{R}^+$). Note that the factor 2 was introduced to simplify arithmetic operations. We can then model the execution time of BERT as

$$t_{BERT}(N) = \underbrace{\frac{N}{2} \cdot 2C}_{\text{1st tree level}} + \underbrace{\frac{N}{4} \cdot 2 \cdot 2C}_{\text{2nd tree level}} + \cdots \tag{35}$$

$$= N \log_2(N) C \tag{36}$$

$$\equiv NMC. \tag{37}$$

Here, the execution time was modeled to double on each consecutive tree level due to increasing number of observations per batch – i.e., assuming compute-bound batch-effect correction of the underlying algorithms ComBat and limma.

Constant Correction Time If instead the overheads (e.g., function calls, type casting) dominate the execution time for one pairwise correction step, it is safe to approximate the required time per correction as constant C' at each tree level, where $C' \in \mathbb{R}^+$. Employing the closed-form solution for the partial sum of the geometric series, the total execution time of BERT can then be modeled as

$$t'_{BERT}(N) = \left(\frac{N}{2} + \frac{N}{4} + \cdots\right) \cdot C' \tag{38}$$

$$= NC' \cdot \sum_{k=1}^{M} \frac{1}{2^k} \tag{39}$$

$$= NC' \cdot \left(1 - \frac{1}{2^M}\right). \tag{40}$$

It follows from Eq. (40) that BERT then exhibits linear time complexity $\mathcal{O}(N)$, which represents the optimum achievable with the underlying batch-effect correction algorithms. Experiments in the main manuscript confirm this linear scaling for up to 64 batches (cf. Fig. 3 D.1), which highlights the mathematically derived suitability of BERT for large data.

Supplementary References

[1] W. Evan Johnson, Cheng Li, and Ariel Rabinovic. "Adjusting batch effects in microarray expression data using empirical Bayes methods". In: *Biostatistics* 8.1 (Apr. 2006), pp. 118–127. ISSN: 1465-4644. DOI: 10.1093/biostatistics/kxj037.

[2] Matthew E. Ritchie et al. "limma powers differential expression analyses for RNA-sequencing and microarray studies". In: *Nucleic Acids Research* 43.7 (Jan. 2015), e47–e47. ISSN: 0305-1048. DOI: 10.1093/nar/gkv007.

[3] Aaron Meurer et al. "SymPy: symbolic computing in Python". In: *PeerJ Computer Science* 3 (Jan. 2017), e103. ISSN: 2376-5992. DOI: 10.7717/peerj-cs.103.

[4] Anna Pursiheimo et al. "Optimization of Statistical Methods Impact on Quantitative Proteomics Data". In: *Journal of Proteome Research* 14.10 (Sept. 2015), pp. 4118–4126. ISSN: 1535-3907. DOI: 10.1021/acs.jproteome.5b00183.

[5] Yannis Schumann, Antonia Gocke, and Julia E. Neumann. "Computational Methods for Data Integration and Imputation of Missing Values in Omics Datasets". In: *PROTEOMICS* (Dec. 2024). ISSN: 1615-9861. DOI: 10.1002/pmic.202400100.

[6] Roderick J. A. Little and Donald B. Rubin. *Introduction*. Aug. 2002. DOI: 10.1002/9781119013563.ch1.

[7] Runmin Wei et al. "Missing Value Imputation Approach for Mass Spectrometry-based Metabolomics Data". In: *Scientific Reports* 8.1 (Jan. 2018). ISSN: 2045-2322. DOI: 10.1038/s41598-017-19120-0.

[8] Charlotte Soneson, Sarah Gerster, and Mauro Delorenzi. "Batch Effect Confounding Leads to Strong Bias in Performance Estimates Obtained by Cross-Validation". In: *PLoS ONE* 9.6 (June 2014). Ed. by Shu-Dong Zhang, e100335. ISSN: 1932-6203. DOI: 10.1371/journal.pone.0100335.

[9] Yannis Schumann, Julia E. Neumann, and Philipp Neumann. "Robust classification using average correlations as features (ACF)". In: *BMC Bioinformatics* 24.1 (Mar. 2023). ISSN: 1471-2105. DOI: 10.1186/s12859-023-05224-0.

[10] Yannis Schumann et al. "Morphology-based molecular classification of spinal cord ependymomas using deep neural networks". In: *Brain Pathology* 34.5 (Jan. 2024). ISSN: 1750-3639. DOI: 10.1111/bpa.13239.

[11] J Luo et al. "A comparison of batch effect removal methods for enhancement of prediction performance using MAQC-II microarray gene expression data". In: *The Pharmacogenomics Journal* 10.4 (July 2010), pp. 278–291. ISSN: 1473-1150. DOI: 10.1038/tpj.2010.57.

[12] Pierre Bellec et al. "The Neuro Bureau ADHD-200 Preprocessed repository". In: *NeuroImage* 144 (Jan. 2017), pp. 275–286. ISSN: 1053-8119. DOI: 10.1016/j.neuroimage.2016.06.034.

[13] Elena Sokolova et al. "Statistical Evidence Suggests that Inattention Drives Hyperactivity/Impulsivity in Attention Deficit-Hyperactivity Disorder". In: *PLOS ONE* 11.10 (Oct. 2016). Ed. by Hanna Christiansen, e0165120. ISSN: 1932-6203. DOI: 10.1371/journal.pone.0165120.
