# Supplementary material for: High performance data integration for large-scale analyses of incomplete Omic profiles using Batch-Effect Reduction Trees (BERT)
Source: Nat Commun. 2025 Aug 2;16:7104. doi: 10.1038/s41467-025-62237-4 (PMC12318123; doi:10.1038/s41467-025-62237-4)
Supplement: Supplementary file 2 — Reporting Summary [file 41467_2025_62237_MOESM2_ESM.pdf]

nature portfolio

Corresponding author(s): Yannis Schumann

Last updated by author(s): Jun 11, 2025

Reporting Summary

Nature Portfolio wishes to improve the reproducibility of the work that we publish. This form provides structure for consistency and transparency in reporting. For further information on Nature Portfolio policies, see our Editorial Policies and the Editorial Policy Checklist.

Statistics

For all statistical analyses, confirm that the following items are present in the figure legend, table legend, main text, or Methods section.

n/a	Confirmed	
☐	☒	The exact sample size (*n*) for each experimental group/condition, given as a discrete number and unit of measurement
☐	☒	A statement on whether measurements were taken from distinct samples or whether the same sample was measured repeatedly
☐	☒	The statistical test(s) used AND whether they are one- or two-sided<br>*Only common tests should be described solely by name; describe more complex techniques in the Methods section.*
☐	☒	A description of all covariates tested
☐	☒	A description of any assumptions or corrections, such as tests of normality and adjustment for multiple comparisons
☐	☒	A full description of the statistical parameters including central tendency (e.g. means) or other basic estimates (e.g. regression coefficient) AND variation (e.g. standard deviation) or associated estimates of uncertainty (e.g. confidence intervals)
☐	☒	For null hypothesis testing, the test statistic (e.g. *F*, *t*, *r*) with confidence intervals, effect sizes, degrees of freedom and *P* value noted<br>*Give P values as exact values whenever suitable.*
☐	☒	For Bayesian analysis, information on the choice of priors and Markov chain Monte Carlo settings
☐	☒	For hierarchical and complex designs, identification of the appropriate level for tests and full reporting of outcomes
☐	☒	Estimates of effect sizes (e.g. Cohen's *d*, Pearson's *r*), indicating how they were calculated

Our web collection on statistics for biologists contains articles on many of the points above.

Software and code

Policy information about availability of computer code

Data collection	The BERT algorithm is publicly available via https://github.com/HSU-HPC/BERT
Data analysis	All experiments have been conducted on nodes of the cluster maxwell at DESY. If not explicitly specified otherwise, the experiments in this work have been conducted using the latest versions of BERT and HarmonizR.<br><br>For our experiments, we used R version 4.4.2 (2024-10-31) with BERT 1.3 and HarmonizR 1.6. For data analysis and classification experiments, we used Python 3.12.10 with scikit-learn 1.6.1, matplotlib 3.10.1, seaborn 0.13.2, pandas 2.2.3 and numpy 2.2.4. We provide a reproducible environment via a container definition file (apptainer) in the GitHub (https://gitlab.desy.de/yannis.schumann/bert_experiments) repository for experiments.

For manuscripts utilizing custom algorithms or software that are central to the research but not yet described in published literature, software must be made available to editors and reviewers. We strongly encourage code deposition in a community repository (e.g. GitHub). See the Nature Portfolio guidelines for submitting code & software for further information.

Data

Policy information about availability of data

All manuscripts must include a data availability statement. This statement should provide the following information, where applicable:

- Accession codes, unique identifiers, or web links for publicly available datasets
- A description of any restrictions on data availability
- For clinical datasets or third party data, please ensure that the statement adheres to our policy

Source data are provided with this paper. Study results are based on third party data, which is publicly available as detailed in the respective original publications. The proteomic data by Petralia et al. used in this study are available in the Proteomics Data Commons database under accession code PDC000180 [https://pdc.cancer.gov/pdc/study/PDC000180]. The proteomic data by Krug et al. used in this study are available in the Proteomics Data Commons database under accession code PDC000120 [https://pdc.cancer.gov/pdc/study/PDC000120]. The proteomic data by Nusinow et al. used in this study are available in the MassIVE database under accession code MSV000085836 [https://massive.ucsd.edu/ProteoSAFe/dataset.jsp?task=02cd1b6a7c674f3ebdbed300b5d9aa57]. The proteomic data by Voss and Schlumbohm et al. used in this study are available in the PRIDE database under accession code PXD027467 [https://www.ebi.ac.uk/pride/archive/projects/PXD027467]. The simulated proteomic data by Goh and Wong are available directly from their publication [https://doi.org/10.1186/s12864-017-3490-3]. The transcriptomics data used in this study are available in the GEO database under accession codes GSE18520 [https://www.ncbi.nlm.nih.gov/geo/query/acc.cgi?acc=GSE18520], GSE66957 [https://www.ncbi.nlm.nih.gov/geo/query/acc.cgi?acc=GSE66957] and GSE69428 [https://www.ncbi.nlm.nih.gov/geo/query/acc.cgi?acc=GSE69428]. The metabolomic data by Wehrens et al. used in this study are available via GitHub [https://github.com/rwehrens/BatchCorrMetabolomics/tree/master/data]. Any additional data processing is described in Sec. Methods. We further publish an extended repository (https://gitlab.desy.de/yannis.schumann/bert_experiments) with all scripts for simulations, omic data analyses and publication-ready plots, where all elements of the study are encapsulated into appropriate make targets. Furthermore, we also provide the definition file for a container in which we executed all our experiments to allow for maximum reproducibility. The BERT software is publicly available as reported in Sec. Code Availability.

Code Availability

The BERT algorithm is publicly available as R package via Bioconductor (https://www.bioconductor.org/packages/release/bioc/html/BERT.html), as well as via https://github.com/HSU-HPC/BERT and https://doi.org/10.5281/zenodo.15607757. The documentation includes extensive installation instructions and working examples. User support is provided via the Bioconductor forum and the GitHub issue section.

Research involving human participants, their data, or biological material

Policy information about studies with human participants or human data. See also policy information about sex, gender (identity/presentation), and sexual orientation and race, ethnicity and racism.

Reporting on sex and gender	N/A
Reporting on race, ethnicity, or other socially relevant groupings	N/A
Population characteristics	N/A
Recruitment	N/A
Ethics oversight	N/A

Note that full information on the approval of the study protocol must also be provided in the manuscript.

Field-specific reporting

Please select the one below that is the best fit for your research. If you are not sure, read the appropriate sections before making your selection.

☒ Life sciences ☐ Behavioural & social sciences ☐ Ecological, evolutionary & environmental sciences

For a reference copy of the document with all sections, see nature.com/documents/nr-reporting-summary-flat.pdf

Life sciences study design

All studies must disclose on these points even when the disclosure is negative.

Sample size	N/A (reanalysis of third-party data)
Data exclusions	N/A (reanalysis of third-party data)
Replication	N/A (reanalysis of third-party data)
Randomization	N/A (reanalysis of third-party data)
Blinding	N/A (reanalysis of third-party data)

Reporting for specific materials, systems and methods

We require information from authors about some types of materials, experimental systems and methods used in many studies. Here, indicate whether each material, system or method listed is relevant to your study. If you are not sure if a list item applies to your research, read the appropriate section before selecting a response.

Materials & experimental systems

n/a	Involved in the study
☒	☐ Antibodies
☒	☐ Eukaryotic cell lines
☒	☐ Palaeontology and archaeology
☒	☐ Animals and other organisms
☒	☐ Clinical data
☒	☐ Dual use research of concern
☒	☐ Plants

Methods

n/a	Involved in the study
☒	☐ ChIP-seq
☒	☐ Flow cytometry
☒	☐ MRI-based neuroimaging

Plants

Seed stocks	N/A
Novel plant genotypes	N/A
Authentication	N/A
